# Multiple Joint Osteonecrosis in a Patient on Long-term Intranasal Corticosteroids

**DOI:** 10.5435/JAAOSGlobal-D-20-00095

**Published:** 2020-11-12

**Authors:** Mohamed A. Yousef, David C. Ayers

**Affiliations:** From the Department of Orthopedic Surgery, University of Massachusetts Medical School, Worcester, MA (Dr. Yousef and Dr. Ayers), and the Department of Orthopaedic Surgery, Sohag University, Sohag, Egypt (Dr. Yousef).

## Abstract

We present the first report of bilateral knee and left ankle osteonecrosis in a 58-year-old female patient on long-term intranasal corticosteroids. Initially, our patient presented with progressive disabling knee pain with normal radiographs. The patient was presumed to have mild degenerative joint disease; therefore, she was treated conservatively. Then, the patient developed severe left ankle pain, and she was thought to have L5/S1 radiculopathy; therefore, she underwent epidural steroid injection that did not provide any benefit. However, extensive bilateral osteonecrosis of the medial tibial plateau in addition to osteonecrosis of the talus bone of left ankle were later diagnosed by MRI. The patient underwent staged bilateral total knee arthroplasty. In conclusion, the diagnosis of osteonecrosis might be challenging because of overlapping clinical presentation with other disorders particularly in the early stage of the disease with normal radiographs. Therefore, a high index of suspicion and thorough history with supplemental MRI imaging are essential for the assessment of patients presented with atypical refractory joint pain particularly in the presence of risk factors.

Osteonecrosis (ON) is a devastating progressive disease that results in debilitating joint degeneration with functional impairment.^1^ The femoral head is the most common affected site, followed by the humeral head and knee.^1^ ON of the knee represents 10% of all patients with ON and typically affects the medial femoral condyle.^2^ Three distinct types of knee ON were described: idiopathic or spontaneous, secondary that occurs in association with identifiable risk factor, and postarthroscopic.^3^ The diagnosis of ON is often challenging and delayed as well because of overlapping clinical presentation with other conditions. Despite the well-known safety of the intranasal corticosteroids, we present the first report of bilateral ON of the medial tibial plateau and left ankle ON in a 58-year-old female patient on long-term intranasal corticosteroids. The patient was informed that data concerning the case would be submitted for publication, and she provided consent.

## Case Report

**Figure 1:**
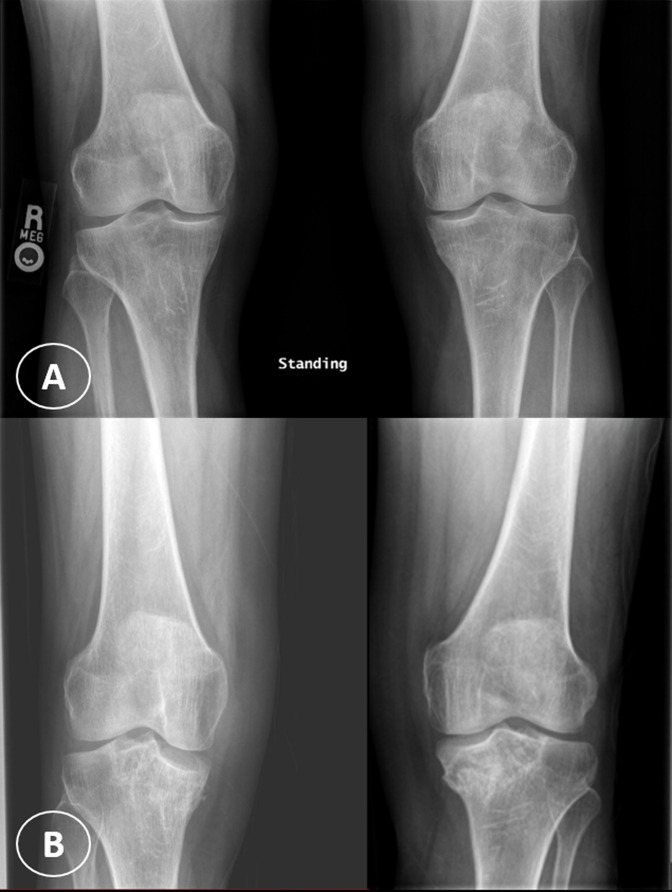
Anteroposterior plain radiograph of both knees done at the initial presentation with normal appearance (**A**) and at 3 months afterward (**B**) demonstrating bilateral subchondral sclerotic lesions and collapse of the medial tibial plateau more severe in the right knee.

**Figure 2:**
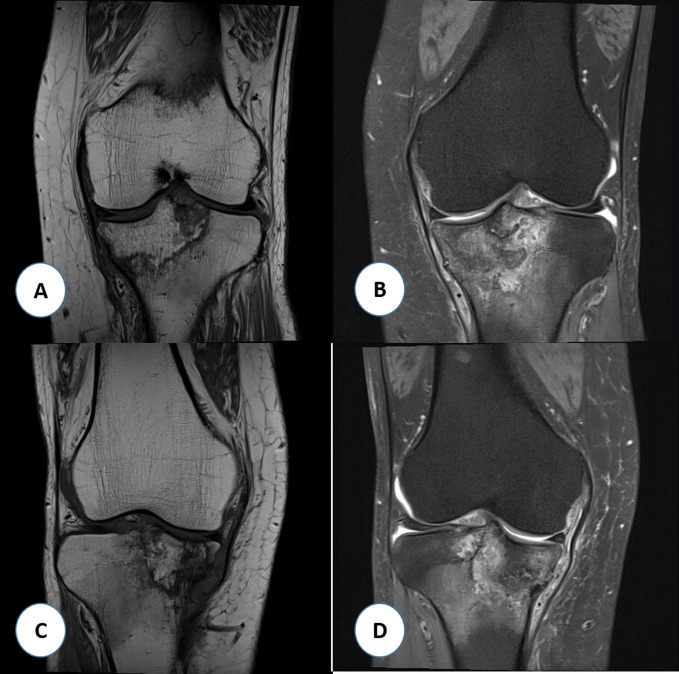
T1-weighted (**A**) and T2-weighted (**B**) MRI images of the left knee demonstrating a large area of acute bone marrow edema with abnormal signal intensity in the medial tibial plateau and irregularly contoured outline between the viable and necrotic bones. T1-weighted (**C**) and T2-weighted (**D**) MRI images of the right knee showing similar findings but more extensive to the left knee with partial collapse of the medial tibial plateau.

A 58-year-old female patient presented to the outpatient clinic complaining of a gradual onset of constant left knee pain of a 9-week duration after several visits to her primary care provider as well as an orthopaedic surgeon without improvement. No history of trauma was noted, and her pain was localized to the medial and anterior aspects of the knee. On examination, the patient walked with antalgic gait. Localized medial tenderness was elicited. No swelling nor signs of internal derangement were noted. She was able to extend to 0° and flex to 135°. Knee radiographs were negative with maintained joint space and without evidence of fracture or notable osteoarthritis (Figure 1, A). The patient was presumed to have mild degenerative joint disease; therefore, she was prescribed naproxen and was sent to physical therapy for 4 weeks. On the follow-up, the pain did not improve, and she started to complain of similar pain affecting her right knee. MRI of the left knee was done that showed a large area of acute bone marrow edema with abnormal signal intensity in the medial tibial plateau measuring 6.5 × 5.2 cm, suggestive of insufficiency fracture (Figure 2, A and B). The knee was placed in a hinged brace, and the patient started to use a walker for protected weight-bearing. Medial history and medications were reviewed. She had hypothyroidism, hypertension, depression, and allergic rhinitis. Her medication included levothyroxine, escitalopram, hydrochlorothiazide, and intranasal corticosteroids (fluticasone propionate [Flonase] two puffs each nostril twice a day) for several years. She never smoked and often drank 1 to 2 glasses of wine a day.

**Figure 3:**
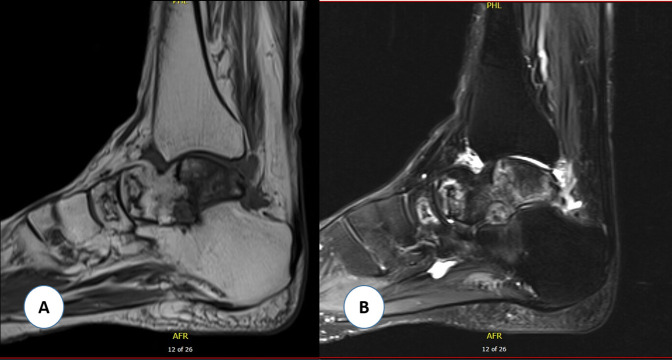
T1-weighted (**A**) and T2-weighted (**B**) MRI images of the left ankle demonstrating area of acute bone marrow edema with abnormal signal intensity affecting the talus and navicular bones.

Because of persistent pain of the right knee, MRI of the right knee was done that showed similar findings but more extensive compared to the left knee with partial collapse of the medial tibial plateau (Figure 2, C and D). A linear T2-hyperintense signal was identified in the medical and lateral meniscus as well but without definite articular surface tear. At that time, hip radiographs were done that showed no evidence of ON, and new knee radiographs showed bilateral subchondral sclerotic lesions, with collapse of the medial tibial plateau more severe in the right knee (Figure 1, B). She was treated conservatively with ibuprofen, gabapentin, diphosphonate (alendronate 70 mg once a week), and physical therapy. At 8 weeks of follow-up, her knee pain had become less intense, but she started to experience disabling pain in her left ankle. Ankle radiographs were negative. She was thought to have L5/S1 left radiculopathy; therefore, she underwent left L5/S1 transforaminal epidural steroid injection under fluoroscopy, which did not provide any benefit. Because of continuing pain, MRI of the left ankle was done that showed extensive ON involving the talus and navicular bones (Figure 3). The patient received additional tests including connective tissue disease workup (complete blood count, erythrocyte sedimentation rate, C-reactive protein, urine analysis, serum creatinine, rheumatoid factor, antinuclear antibody, antidouble stranded DNA, lupus anticoagulant, and anticardiolipin antibodies), coagulation profile (prothrombin time, international normalized ratio, activated partial thromboplastin time, and fibrinogen), and thrombophilia workup (antithrombin, protein C, protein S, factor V Leiden, and factor VIII) to exclude underlying diseases that were negative.

**Figure 4:**
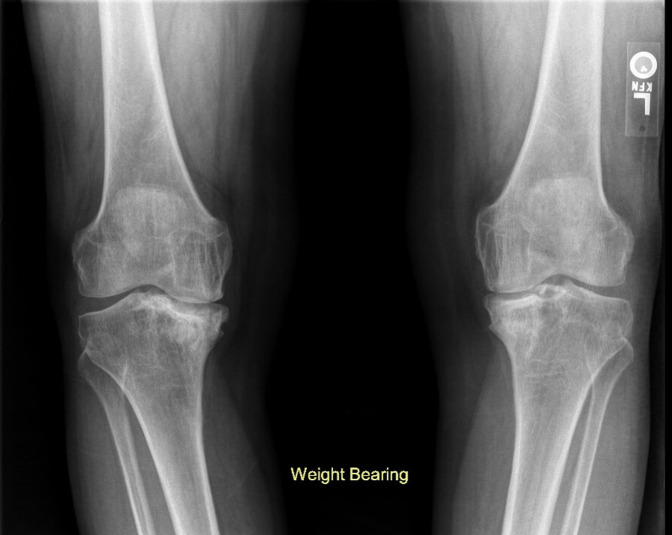
Follow-up anterior-posterior weight-bearing plain radiographs of both knees demonstrating progressive collapse of the medial tibial plateau with varus malalignment and secondary osteoarthritis bilaterally more severe on the right.

**Figure 5:**
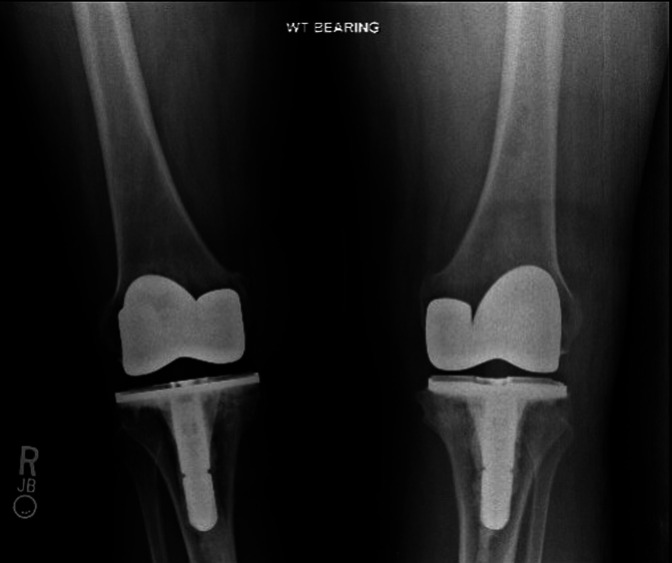
Postoperative anterior-posterior weight-bearing plain radiograph demonstrating bilateral total knee arthroplasty in good position and alignment.

**Figure 6:**
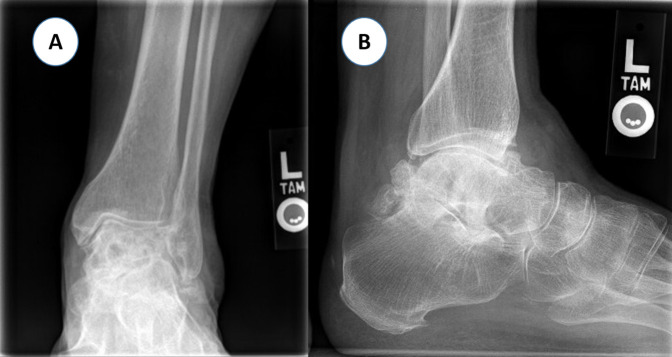
Anteroposterior (**A**) and lateral (**B**) plain radiograph of the left ankle demonstrating extensive mixed area of sclerosis and luscencies within the talus with flattened talar dome, partial talar collapse, and secondary degenerative osteoarthritis of the tibiotalar and subtalar joints.

Follow-up knee radiographs showed progressive collapse of the medial tibial plateau with varus malalignment and secondary osteoarthritis bilaterally. The disease progressed to stage IV according to the Ficat and Arlet classification^3^ (Figure 4) over 2 years. Staged bilateral total knee arthroplasty (TKA) was performed because of progressively worsening knee pain recalcitrant to conservative treatment (Figure 5). Bone histopathology confirmed bone ON. The patient recovered well after TKA, with great improvement of pain and function at the 6-month follow-up. The mean knee injury and osteoarthritis outcome score (KOOS) pain scale improved from 38 before surgery to 83. Similarly, the mean KOOS function in daily living (ADL) scale improved from 37 before surgery to 85. The patient achieved an active range of knee motion from 0 to 128° of flexion, with no extension lag. The left ankle was treated conservatively with solid ankle-foot orthosis to provide some comfort and to postpone the ankle surgery until she recovers from the TKA. The ankle ON progressed with talar collapse and secondary degenerative arthritis of the tibiotalar and subtalar joints (Figure 6).

## Discussion

Ahlback and colleagues were the first to describe the spontaneous type of knee ON in 1968.^4^ It is the most common form of knee ON.^3^ Its exact etiology is still unclear; however, it is thought that vascular insufficiency or traumatic event might be implicated in the necrosis of subchondral bone with subsequent development of articular surface collapse and secondary osteoarthritis.^5^ This clinical entity is often restricted to the medial femoral condyle with particular unilateral affection of the epiphysis and without other joint involvement or associated comorbidity. It affects older individuals older than 55 years.^3^ While secondary knee ON usually affects the epiphysis, metaphysis, and even diaphysis, are found in patients younger than 55 years. It is characterized by the presence of bilateral multiple lesions that affect the hip in 90% of the patients.^3^ It occurs in association with steroid use, alcoholism, connective tissue disease, sickle cell disease, thrombophilia, and in patients with organ transplantation.^1,3^

Patients with allergic rhinitis represent approximately 10% to 30% of the general population, and the prevalence tends to increase progressively across the world.^6^ Although the use of intranasal corticosteroids is considered safe, occasional adverse events have been reported such as adrenal suppression, increased intraocular pressure, and growth suppression.^7^ Although it was shown that 90% of patients with knee ON have a history of steroid use, data on the dose, route of administration, and length of treatment that can cause ON are remarkably variable.^8,9^ Although long-term high-dose steroid therapy is a major risk factor for ON,^9^ ON can develop after short-term low-dose therapy,^10^ intra-articular injection,^11^ or inhalational steroid.^12^ It is believed that steroids increase the bone marrow adipocyte cell size with elevation of the intraosseous pressure and subsequent impairment of the blood flow.^13,14^ The combined use of alcohol and steroids is shown to have an additive effect in causing ON.^15^ We feel that despite the low systemic bioavailability of the intranasal corticosteroids, they can cause ON in the presence of increased alcohol intake.

Patients usually present with gradual onset of severe medial knee pain that is often worse at night or with weight-bearing.^3^ The diagnosis of knee ON is often challenging because it is assumed that the knee pain might be due to meniscal injury or early arthritic changes.^8^ Diagnostic workup including connective tissue screening tests and thrombophilia tests can be considered in patients with ON.^16,17^ However, their role in patient management is not clear. The plain radiographs are completely normal initially; therefore, high index of clinical suspicion and thorough history are important to identify possible risk factors. Later on, radiographs show subchondral radiolucent areas and eventual articular surface collapse with arthritic changes as the disease progresses.^3^ At early stage disease, MRI is the modality of choice to establish the diagnosis.^18^ It provides detailed information about the extension of the lesion and any other associated intra-articular lesions. MRI is also an important screening imaging tool in high-risk patients, regardless of the symptoms.^14^ Although bone scintigraphy is an effective diagnostic tool, it has a much lower sensitivity of 64% compared with 100% reported using MRI beside its limited value in patients with multifocal disease.^18^ The duration from the start of symptoms to the time of diagnosis is variable ranging from two weeks to 24 months.^6^ In our patient, the diagnosis was made 13 weeks after the pain started. Early diagnosis is important to avoid mistreatment and to avoid or delay the need for arthroplasty especially in younger patients.

Although it is difficult to differentiate between spontaneous and secondary knee ON based on the clinical presentation, demographic and radiological findings can help identify the type of knee ON. A relatively young patient with multiple joints involvement and underlying risk factors, as is the case with our patient, makes the diagnosis of secondary ON more likely. In addition, MRI findings are useful to identify the type of knee ON particularly those with notably larger lesions in patients with secondary ON and with the presence of irregular well-demarcated line between the necrotic and viable bone.^1^ Only few cases of bilateral knee ON have been reported in the literature with affection of the patella^12,19^ and the lateral femoral condyle.^20,21^ The lesions in our case were localized to the medial tibial plateau bilaterally, which is unusual. All cases shared the same risk factor of steroid use that varied from the typical oral form^20^ to the inhaled form.^12^ In our case, intranasal corticosteroids were responsible for ON.

The conservative treatment of knee ON, which included protected weight-bearing and the use of analgesics and diphosphonate, was unsuccessful. Because the disease continued to progress rapidly, joint-preserving procedures such as core decompression and/or impacted bone grafting were not an option for this patient. Because of extensive bone involvement, we proceeded with TKA, which provides excellent results–comparable with those reported in patients with osteoarthritis--in patients with ON, rather than unicompartmental arthroplasty.^22^ Our patient's KOOS pain and function scores improved greatly after TKA. The management of talus ON is often challenging with suboptimal outcome in large percentage of patients.^23^ The management options include conservative treatment with protected weight-bearing or surgical treatment with core decompression, vascularized bone grafting, total talar prosthesis, or salvage procedure.^23^ The surgical treatment for our patient would have been quite extensive requiring a salvage procedure because of the extensive lesions affection the talus and navicular bones.

In conclusion, bone ON is an unusual, debilitating disease that necessitates early diagnosis. ON should be considered in the differential diagnosis of severe atypical refractory joint pain particularly in the presence of identified risk factors. The diagnosis might be challenging because of overlapping clinical presentation with other disorders particularly in the early stage of the disease with normal radiographs. Therefore, a high index of suspicion and thorough history with supplemental MRI imaging are essential for the diagnosis of such condition to avoid mistreatment or delays in treatment especially in younger patients.
